# Pathogenesis-Guided Biomarker Assessment: A Shift in Prostate Cancer Diagnostics

**DOI:** 10.3390/ijms262411786

**Published:** 2025-12-05

**Authors:** Jessica M. Logan, Victoria Malone, John J. O’Leary, Doug A. Brooks

**Affiliations:** 1 Clinical and Health Sciences, University of South Australia, Adelaide 5000, Australia; 2 Department of Pathology, The Coombe Women and Infants University Hospital, D08 XW7X Dublin, Ireland; 3 Department of Histopathology, Trinity College Dublin, D02 PN40 Dublin, Ireland

**Keywords:** prostate cancer, biomarkers, diagnosis, pathogenesis

## Abstract

Despite prostate cancer being one of the most common malignancies in men, its pathological diagnosis remains plagued by inter-observer variability and diagnostic ambiguity. Traditional morphological assessment and currently available biomarkers such as PSA (Prostate-Specific Antigen), AMACR (Alpha methylacyl CoA racemase), and p63 suffer from poor specificity and clinical reliability. In this review, we present a pathogenesis-guided biomarker discovery strategy that led to the development of a clinically validated biomarker panel—Appl-1, Sortilin, and Syndecan-1. These biomarkers, which reflect fundamental biological processes within the endosome–lysosome system, offer improved diagnostic precision and prognostic utility for patients with prostate cancer. This review discusses the rationale behind their discovery, the multidisciplinary approach that enabled it, the evidence supporting their use, and their implementation in U.S. clinical practice as a lab-developed test (LDT). We propose this approach as a new diagnostic standard that bridges mechanistic insight with real-world application.

## 1. Introduction

Prostate cancer is a major global health challenge, with over 1.4 million men diagnosed annually and an incidence that is projected to double by 2040 [1,2]. This growing burden strains clinical pathology service provision, with the related global healthcare costs already exceeding USD 6 trillion annually [3]. Despite advancements in treatment, the disease still causes over 375,000 deaths worldwide each year. Early and accurate diagnosis is critical for improving survival rates through timely and appropriate intervention [4]. Active surveillance (AS) is an approach for managing prostate cancer by clinical observation, biochemical testing and the analysis of repeat biopsies. Patients with prostate cancer who are suitable for AS are stratified as low-risk, and, for example, in Australia this represents around 13–30% of all men diagnosed with prostate cancer each year [5]. Regrettably, in the first 2 years post prostate cancer diagnosis, 35% of men on AS will require radical treatment intervention, and this increases to 59% within 5 years [5]. Nearly one-third of patients with prostate cancer experience biochemical recurrence (BCR) after primary definitive treatment, while similar numbers will experience a metastatic event within 10 years of diagnosis [6,7]. There is a defined need for technological advancements to improve methods to reliably stratify low-risk and high-risk disease patients to maximize early appropriate treatment interventions, while balancing the morbidity caused, to ultimately improve outcomes and reduce the socioeconomic burden of prostate cancer. Therefore, there is an urgent need for innovative technologies to enhance pathology assessment and patient management, to improve treatment outcomes.

## 2. Overcoming the Limitations of Gleason Grading in Prostate Cancer Assessment

Prostate cancer diagnosis primarily relies on histopathological examination of needle biopsy specimens stained with hematoxylin and eosin (H&E) [4,5,8,9,10]. The diagnostic and prognostic standard is the Gleason grading system, which evaluates glandular architectural patterns and categorizes tumors based on their degree of differentiation [5,8,9]. Gleason patterns range from well-formed glands (pattern 3) to fused or poorly formed glands (pattern 4), to sheets of undifferentiated cells, necrosis, or single-cell infiltration (pattern 5) [5,8,9]. The sum of the two most prevalent patterns gives rise to the Gleason score, which is subsequently translated into the International Society of Urological Pathology (ISUP) Grade Groups 1 through 5 [5,8,9].

While the current prostate cancer grading system is clinically entrenched, it remains subjective, being dependent upon the morphological complexity of the tissue sample and the ability of the observer to interpret the pathology. In essence, routine H&E staining of tissue samples cannot depict the critical pathological changes driving the morphological variations, and this results in inconsistent Gleason grading and occasionally misdiagnosis [4,5,8,9,10,11,12,13]. Attempts to address the issues with Gleason grading, such as the adoption of ISUP grade grouping, have somewhat improved classifications, but still result in a substantial number of patients being inaccurately graded, leading to suboptimal therapeutic decision-making [4,5,8,9,10,11,12,13,14,15,16,17,18]. Morphological assessment is particularly prone to variability when analyzing tumor architecture, where distinguishing between benign, transition zones and malignant regions becomes ambiguous. Substantial inter-observer variability is common, especially in distinguishing between patterns 3 and 4 or identifying complex structures like cribriform glands and intraductal carcinoma of the prostate (IDCP) [1,2,3,4,5,8,9,10,11]. Significant histological variants, such as cribriform, glomeruloid and IDCP, further complicate grading [5,8,9]. Some pathological subtypes, including adenoid cystic (basal cell) carcinoma and small-cell carcinoma, fall outside the Gleason system, adding to this diagnostic complexity [5,8,9]. These distinctions are crucial as patients with ISUP Grade Group 2 (Gleason score 3 + 4) often undergo active surveillance, while those with Grade Group 3 (Gleason score 4 + 3) are directed toward radical intervention. Misclassification may result in overtreatment or undertreatment, with serious implications for outcomes and quality of life [4,5,8,9,10,11,12,13,14,15,16,17,18]. To improve grading accuracy, pathologists frequently employ adjunct immunohistochemical (IHC) stains, such as AMACR (Alpha-methylacyl-CoA racemase; p504S), PSA (prostate-specific antigen), PSMA (prostate-specific membrane antigen), p63, and high-molecular-weight cytokeratin (34βE12) [13,14,15,16]. However, these markers have inherent limitations [16,19,20]. AMACR, while overexpressed in cancer, is not consistently detected in all malignancies and is subject to variability due to metabolic conditions. For example, AMACR’s variability is influenced by the altered metabolism of cancer cells, including factors such as resource availability, stage of growth, and metastatic potential, all of which restrict its clinical utility. PSA and PSMA provide a degree of organ specificity but do not contribute meaningfully to grading. In current clinical practice, basal cell markers such as high-molecular-weight cytokeratins (34βE12, cytokeratin 5/6), p63, or a combination of these, can be utilized to visualize the basal cell layer. The absence of basal cells often serves as an indicator of prostate cancer or alternatively age-related changes, yet this negative labeling strategy does not remove grading interpretation or detection difficulties for tumors. Consequently, basal cell markers help distinguish benign from malignant glands by their absence in the vicinity of prostate cancer but offer no insight into tumor biology or its progression.

Other markers, including prostate-specific markers such as PCA3 (prostate cancer antigen 3), NKX3.1 and PSAP (prostate-specific acid phosphatase), have also been explored but also exhibit sensitivity and specificity issues that constrain their practical application [19,20,21]. While additional markers like Ki67 and p16 have been investigated in this setting, they are limited in their ability to assist in ISUP grade group assignment or reliably predict prognosis for patients with prostate cancer. The proposed utility of existing biomarkers for prostate histopathology assessment has been detailed extensively by Kielb et al. [22]. The clinical relevance and proposed utility of these biomarkers for prostate histopathology assessment have been detailed extensively in Table 1. This diagnostic ambiguity highlights a significant unmet need for novel, robust biomarkers that not only assist in detection but also align with disease pathogenesis and reliably inform tumor grading and prognosis [17,18,23,24,25,26,27,28,29,30,31].

## 3. New Approaches to Biomarker Discovery

As one of the most prevalent malignancies in men and a leading cause of cancer-related mortality, prostate cancer imposes a substantial patient and financial burden on global healthcare systems [17,18,23,24,25,26,27,28,29,30,31]. The need to develop effective prostate cancer diagnostics for clinical practice necessitated a novel and innovative multidisciplinary approach. To achieve translational success, we assembled a multidisciplinary team comprising cancer biologists, histopathologists, clinicians, consumer advocates, implementation scientists, bioinformaticians, antibody engineers, and industry partners to develop high-sensitivity and high-specificity tissue diagnostics for prostate cancer [17,18,23,24,25,26,27,28,29,30,31]. We also needed to change the approach to biomarker discovery and focus on the fundamental biology and primary pathogenesis of the cancer. Traditional biomarker discovery has largely relied on cell line and animal model systems, which often fail to replicate the complexity and heterogeneity of human disease. Many studies have focused on identifying individual or random combinations of targets, which often fail in clinical translation due to variability in datasets and biological inconsistencies [17,18,23,24,25,26,27,28,29,30,31]. Recognizing the complexity of prostate cancer and the limitations of conventional tools, we adopted a biomarker discovery strategy anchored in the endosome–lysosome system—a pathway deeply implicated in oncogenesis, receptor signaling, and intracellular trafficking [17,18,23,24,25,26,27,28,29,30,31]. In contrast to previous strategies, this novel integrated approach examined critical interconnected elements of the endosome–lysosome system as these organelles provide a framework that is intrinsically linked to cancer hallmarks [17,18,23,24,25,26,27,28,29,30,31]. By leveraging a systematic gene and protein bioinformatics analysis, a functionally interconnected biomarker network was identified within this framework, rather than isolated molecular targets. This combinatorial strategy helped to mitigate dataset variability among biobanks and sampling discrepancies, including variations in tumor versus non-malignant tissue proportions and inconsistencies between gene and protein expression data [17,18,23,24,25,26,27,28,29,30,31]. This analysis was used to generate a potential pathogenic map, embedded within the endosome–lysosome network.

Following bioinformatics-guided selection, a panel of potential biomarkers was validated by protein IHC analysis to select candidates that consistently depicted the primary pathogenesis in patient tissue. The tissue-specific protein data was then used to confirm and refine the pathogenic map. Mechanistic cell biology studies further confirmed these candidates, and established biomarkers indicative of key regulatory points in prostate cancer progression [17,18,23,24,25,26,27,28,29,30,31]. Recognizing the necessity for high-specificity reagents, ISO 9001-standard monoclonal antibodies were developed to target multiple unique linear sequence epitopes on selected biomarkers [17,18,23,24,25,26,27,28,29,30,31]. Only epitopes consistently recognizing specific aspects of the cancer pathogenesis in patient tissue samples were selected for further development.

Stringent technical validation of the reagents was conducted by immunohistochemistry (IHC) testing across multiple clinical platform technologies in a standardized histology facility. This rigorous process was designed to facilitate the seamless translation of our findings into clinical practice and resulted in the identification of three candidate biomarkers with high specificity/sensitivity in prostate cancer tissue samples [17,18,23,24,25,26,27,28,29,30,31]. Cross-validation across independent, highly annotated global biobanks further confirmed the clinical utility of these biomarkers, enabling the establishment of appropriate claims for clinical implementation and product development [17,18,23,24,25,26,27,28,29,30,31]. This novel approach enabled the identification of biomarkers that precisely define the primary pathogenesis in prostate cancer tissue. By integrating advanced bioinformatics, pathway analysis, rigorous validation protocols, and employing dedicated clinical translation strategies, this approach establishes a new benchmark for biomarker discovery, which aligns with enhanced prostate cancer detection and prognosis that can improve patient management [17,18,23,24,25,26,27,28,29,30,31].

**Figure 1:**
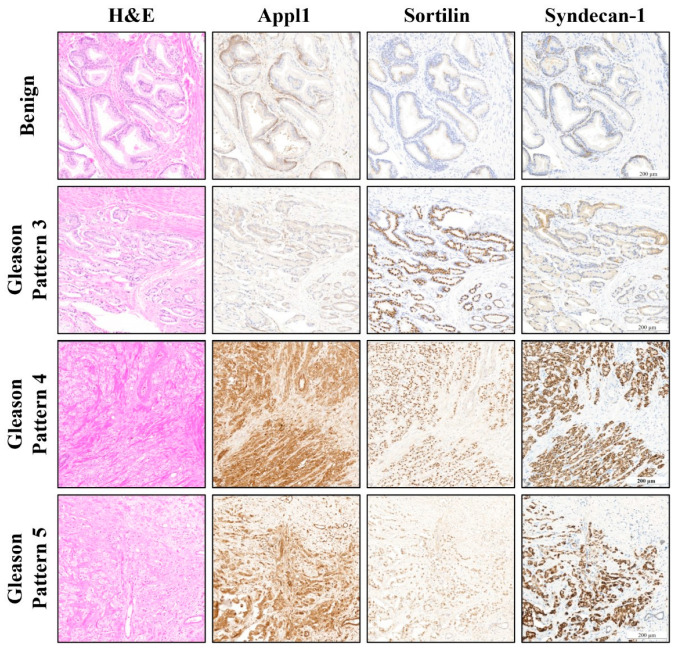
Comparative histological and immunohistochemical analysis of prostate tissue. H&E illustrates classic benign and Gleason pattern 3, 4 and 5 morphologies. Appl-1 labeling (brown) clearly distinguishes the basal cell layer and secretory epithelial cells of benign prostate tissue and shows specific changes in biomarker distribution for benign and cancer tissue. Gleason pattern 3 has a specific increase in Appl-1 cancer cell labeling. Gleason pattern 4 and 5 progression to advanced cancer is accompanied by a marked increase in the intensity of Appl-1 biomarker labeling. Sortilin reliably labels Gleason pattern 3 glands with a polarized granular labeling pattern (brown), supporting the interpretation of well-formed gland morphology. In higher Gleason patterns, the polarity and intensity of Sortilin labeling is lost. Syndecan-1 clearly distinguishes the basal cell layer and secretory epithelial cells in benign prostate tissue, while Gleason pattern 3 neoplastic events are confirmed by the absence of labeling (brown). Syndecan-1 delineates cancer progression with a marked increase in labeling intensity in advanced cancer (Gleason pattern 4 and 5). Figure generated from prostate cancer digital library [17,24].

Three biomarkers emerged as both mechanistically relevant and technically feasible for accurate prostate cancer diagnosis and prognosis, by IHC analysis on patient tissue samples [8,9,17,18,23,24,25,26,27,28,29,30,31]. Appl1 (Adaptor Protein, Phosphotyrosine interacting with PH domain and Leucine Zipper 1) has functions in early endosome compartmentalization/traffic, adipokine receptor signaling, transcription factor activity and regulatory roles in mitophagy and NRLP3 inflammasome activity. The biomarker technology has >95% sensitivity and specificity for prostate cancer detection. A specific epitope on Appl-1 demonstrated ≥95% sensitivity and specificity for prostate cancer in tissue samples, providing robust diagnostic accuracy while visualizing tumor architecture, volume, and leading edges [18]. Furthermore, Appl-1 distinguishes benign tissue through its basal cell distribution, which transitions to a cytoplasmic pattern in malignant cells, offering a pathophysiological basis for its precise detection of prostate cancer (Figure 1) [18,28]. In addition, Sortilin provided reliable detection of low-grade cancer with the accurate detection of well-formed malignant glands. In contrast, Syndecan-1 detected high-grade cancer with precise labeling of poorly formed malignant glands. This new biomarker technology has 1. high accuracy for prostate cancer detection in tissue samples and clinical utility for accurate diagnosis [18,28]; 2. improved accuracy for Gleason scoring and grade grouping that has clinical utility for patient prognosis [18,28].

This is the first biomarker technology that assists in grading prostate cancer tissue samples. Specific epitopes on the biomarkers, Sortilin (transport, metabolic control, cell survival and signal transduction) and Syndecan-1 (integral membrane protein involved in endosomal uptake of lipids/cytokines/chemokines, and with functional roles in cell proliferation, invasion, angiogenesis, host defense and matrix modeling) enable visualization of the specific pathology in prostate cancer tissue that aligns with the different morphologies used in grading (Figure 1). The combinatorial use of Sortilin and Syndecan-1 therefore has clinical utility in refining grade-specific assignments and can improve prognostic capabilities. Sortilin is characterized by a polarized distribution and increased expression in well-formed gland morphology, but diminishes in higher-grade tumors (Figure 1 [18,28]). Conversely, Syndecan-1 detection predominates in poorly formed gland morphologies and is reduced in lower-grade cancers, creating a biomarker system that directly aligns with Gleason grading [17,18,28].

These biomarkers are intrinsically linked to prostate cancer pathogenesis, offering mechanistic insights into the cancer biology and robust alignment with patient outcomes. Unlike traditional immunohistochemical markers derived from association studies, they define the mechanistic and metabolic milieu of prostate cancer, ultimately improving prognosis and molecular disease classification. The biomarkers (Appl1, Sortilin and Syndecan-1) are applicable across the entire biological continuum of prostate diseases from normal, to atypical hyperplasia, to PIN (prostatic intraepithelial neoplasia) to localized and metastatic carcinoma [18,27,28]. Appl-1 functions in early endosome trafficking and mitophagy regulation and shows a benign-to-malignant cytoplasmic shift, providing clear visualization of tumor boundaries [17,18,28]. Appl1 also has function links to transcription factor activity, involving translocation to the nucleus, interacting with histone deacetylases HDAC 1-3 and Reptin to modulate chromatin structure and facilitate gene expression; therefore, it may be integrally involved in prostate cancer disease progression [46,47]. Sortilin is involved in protein sorting and apoptosis control, and demonstrates polarized glandular expression in Gleason 3 cancers with reduced labeling in higher-grade disease [17,18,28]. Sortilin has also been linked with progranulin degradation to delay disease progression and a castrate-resistant phenotype, supporting its association with low-grade disease [48]. Syndecan-1 regulates extracellular matrix remodeling and cell adhesion; its expression is absent in benign tissue but prominent in Gleason 4 and 5 morphologies, including cribriform and fused glands [17,18,23,24,25,26,27,28,29,30,31]. Similarly, Syndecan-1 has been associated with advanced disease, with several studies linking its expression to poorer progression-free survival and independent prediction of biochemical relapse [49,50,51,52,53]. The integration of clinical pathologist feedback into iterative antibody validation stages enabled real-time optimization of diagnostic performance. This collaborative model ensured the final panel was both biologically grounded and compatible with real-world pathology workflows. See Figure 1 [18,27,28].

In addition to superior visualization of prostate cancer pathology in tissue samples from patients with prostate cancer, the new panel of biomarkers provided clinically meaningful interpretation of the pathogenesis. In a retrospective study published by Logan et al. [17], the biomarker panel using Appl1, Sortilin and Syndecan-1 reclassified nearly half of all cases, and these reclassifications were not cosmetic. Long-term follow-up showed that upgraded patients had significantly increased biochemical recurrence (61% vs. 39%) and clinical relapse (22% vs. 8%) rates over a 10-year period [17]. The biomarkers reliably visualized tumor morphology, confirmed atypical cribriform patterning, and supported differentiation between fused and well-formed glands—precisely the areas where conventional Gleason grading falters. Following this validation, the panel was implemented by Quest Diagnostics as a U.S. lab-developed test. This milestone demonstrates that rigorous, multidisciplinary development can translate biomarker research into standardized, high-impact clinical diagnostics.

This biomarker framework reflects a shift from descriptive to mechanistic diagnostics. Rather than relying on non-specific associations, Appl-1, Sortilin, and Syndecan-1 directly mirror the underlying biological architecture and primary pathogenesis of prostate cancer. This specificity not only supports routine diagnosis and prognosis, but also creates high-quality ground truth for emerging AI and digital pathology platforms. Prospective studies are now underway to evaluate the biomarker panel’s predictive utility in active surveillance cohorts and high-risk populations. Additionally, development of multiplexed IHC platforms and digital scoring algorithms is expected to streamline workflow integration. Finally, this biomarker discovery platform may be extended to other morphologically complex malignancies, such as bladder, colorectal and pancreatic cancers.

## 4. Conclusions

The Appl-1, Sortilin, and Syndecan-1 biomarker panel addresses critical diagnostic challenges in prostate cancer pathology by enhancing the accuracy of detection and reproducibility of Gleason grading and ISUP group assignment. Grounded in tumor biology, validated by multidisciplinary collaboration, and integrated into clinical diagnostics, this panel of biomarkers delivers meaningful improvements to patient stratification and prognostic prediction. It exemplifies how pathogenesis-guided biomarkers can raise the standard of care while enabling the transition to AI-assisted, personalized medicine.

The biomarkers Appl-1, Sortilin, and Syndecan-1 represent a transformative advancement in prostate cancer pathology assessment and are now being integrated into clinical practice in the USA [45]. Compared to traditional biomarkers, this innovative technology delivers significantly enhanced diagnostic accuracy, refined morphological assessments, and more reliable prognostic insights. By precisely visualizing critical aspects of tumor pathology, these biomarkers reduce inter-observer variability, addressing a longstanding challenge in pathology interpretation and offering substantial promise for improving patient outcomes [17,18,23,24,25,26,27,28,29,30,31].

The direct involvement in prostate cancer biology and pathogenesis uniquely positions these biomarkers as ideal candidates for the development of AI-driven digital pathology solutions with high ground truth [17,28]. They are also theranostic molecules, potentially targetable and derived from the same system as bioprocesses and they control the expression and secretion of other prostate markers like PSA. The integration of this technology into clinical practice can enable more accurate diagnoses, potentially reducing unnecessary procedures, while refining grading and prognostic capabilities to support personalized treatment strategies. By aligning with the critical need for precision in clinical pathology assessment, this biomarker technology lays a robust foundation for optimizing patient care and improving treatment outcomes [17,28].

## 5. Patents

Multiple patents—including PCT/AU2014/000612 “Methods for detecting prostate cancer” and PCT/AU2020/050925 “Methods for Confirming Detection and Evaluating the Progression of a Prostate Cancer”—involve the biomarkers reported in this manuscript.

## Figures and Tables

**Table 1:** Clinical utility and validation of prostate cancer biomarkers.

Biomarker	Proposed Utility	Clinical Relevance	Limitations	Validation Status
AMACR (P504S)	histopathological marker of malignancy [13,14,15,16,19,20].	used to differentiate ASAP lesions as malignant as well as IDCP [13,14,15,16,19,20].	utility affected by tumor heterogeneity. no expression in neuroendocrine tumors loss of expression in higher-grade tumors [13,14,15,16,19,20] not prognostic	variable sensitivity and specificity reported (70–90%) [32,33] Used clinically, however no standardized protocol and only used at pathologist’s discretion [13,14,15,16,19,20].
P63 (34βE12)	histopathological marker of basal cells in benign/normal prostatic glands [13,14,15,16,19,20].	used when basal cell layer is incomplete negative labeling strategy [13,14,15,16,19,20].	often used in conjunction with CK 5/6 to overcome poor reactivity used in a cocktail format with AMACR [13,14,15,16,19,20].	Used clinically, however no standardized protocol and only used at pathologist’s discretion [13,14,15,16,19,20].
High-molecular-weight cytokeratin (CK 5/6)	histopathological marker that labels basal cells in benign/normal prostatic glands [13,14,15,16,19,20].	used when basal cell layer is incomplete. negative labeling strategy [13,14,15,16,19,20].	often used in conjunction with CK 5/6 to overcome poor reactivity with some basal cell carcinomas and basal cell heterogeneity. used in a cocktail format with AMACR [13,14,15,16,19,20].	Used clinically, however no standardized protocol and only used at pathologist’s discretion [13,14,15,16,19,20].
PSA	blood based diagnosis and screening [22].	used to identify men who should receive a follow-up diagnosis with a urologist	elevated in other conditions, i.e., prostatitis, forms of exercise. free vs. total PSA. levels effect ability to detect PCa (e.g., low levels > false positives but at high levels > false negatives) [34]. limited to no utility in tissue samples.	high sensitivity but poor specificity. used with variable purpose. guidelines are being reviewed to systematically provide patients’ meaningful access.
PSMA prostate-specific membrane antigen	PMSA-PET-guided imaging for clinical monitoring and diagnostic imaging [35].	Staging and monitoring of metastatic disease [35]. treatment planning and monitoring for recurrence [35]. Beyond its use in primary staging and in BCR, PSMA-PET is becoming increasingly incorporated into the management of advanced PCa [35].	false positives/negatives, variable sensitivity for small tumors, lack of long-term outcome data [35]. 5–10% of cancers do not express PSMA and some have variable expression [35].	important tool in the staging and management of intermediate- to high-risk prostate cancer. guidelines are being reviewed to systematically provide patients meaningful access
PCA3 (prostate cancer antigen 3)	The Progensa PCA3 gene assay measures PCA3 mRNA concentrations in the first void urine collected after DRE [36,37,38]. In 2012, the FDA approved the use of PCA3 to facilitate the decision-making process to re-biopsy men with a previous negative biopsy [36,37,38].	Multiple studies have evaluated the clinical utility of PCA3 in the early detection of PCa and as a prognostic marker in the active surveillance of patients with low risk [36,37,38].	exhibits sensitivity and specificity issues that constrain its clinical utility [36,37,38].	not currently a standard part of prostate cancer screening, e.g., in Australia [36,37,38].
NKX3.1	histopathological marker to help distinguish between prostate adenocarcinoma and benign tissue, and for the diagnosis of mesenchymal chondrosarcoma [39].	negative marker for seminal vesicle involvement [40]	exhibits sensitivity and specificity issues that constrain the clinical utility [39,40].	incorporated in routine tumor screening panels in some laboratories not part of ISUP guidelines
PSAP (prostate-specific acid phosphatase)	used to prove origin of metastatic tumors [41].	exhibits sensitivity and specificity issues that constrain the clinical utility [41].	lack of specificity, PSAP alone is not sufficient for a cancer diagnosis [41].	poor specificity and is no longer used.
Ki67	histopathological marker of proliferation [42].	indicates a higher likelihood of aggressive disease but its prognostic value is contradictory [42].	low inter-observer reproducibility [42].	no established protocol for utility.
p16	has proposed utility as a prognostic marker, target for gene therapy and diagnostics [43,44].	p16 is proposed as a potential biomarker for risk stratification and treatment planning in prostate cancer [43,44].	complex and context-dependent role with inconsistent expression [43].	no established protocol for utility.
Appl1	histopathological marker of malignancy. reports on underlying changes in biology [17,18,23,24,25,26,27,28].	ISUP IHC assisted grading. reliable risk prediction of BCR and CR [17,18,23,24,25,26,27,28].	Independent validation required for widespread adoption.	currently utilized as an LDT in the USA [45].
Sortilin	histopathological marker of malignancy and indicates well-formed gland morphology in prostate adenocarcinoma. reports on underlying changes in biology [17,18,23,24,25,26,27,28].	ISUP IHC assisted grading, HGPIN, IDCP, reliable risk prediction of BCR and CR [17,18,23,24,25,26,27,28].	Independent validation required for widespread adoption.	currently utilized as an LDT in the USA [45]
Syndecan-1	histopathological marker of malignancy and indicates poorly formed gland morphology in prostate adenocarcinoma. reports on underlying changes in biology [17,18,23,24,25,26,27,28].	ISUP IHC assisted grading, HGPIN, IDCP, reliable risk prediction of BCR and CR [17,18,23,24,25,26,27,28].	Independent validation required for widespread adoption.	currently utilized as an LDT in the USA [45].

## Data Availability

No new data were created or analyzed in this study. Data sharing is not applicable to this article.

## Abbreviations

The following abbreviations are used in this manuscript:

IHC	Immunohistochemistry
ISUP	International Society of Urological Pathology
H&E	Haematoxylin and Eosin
